# Pharmacological inactivation of the PI3K p110δ prevents breast tumour progression by targeting cancer cells and macrophages

**DOI:** 10.1038/s41419-018-0717-4

**Published:** 2018-06-07

**Authors:** Evangelia Goulielmaki, Miriam Bermudez-Brito, Margarita Andreou, Niki Tzenaki, Maria Tzardi, Eelco de Bree, Eleftheria Tsentelierou, Antonis Makrigiannakis, Evangelia A. Papakonstanti

**Affiliations:** 1 Department of Biochemistry, School of Medicine, University of Crete, Heraklion, Greece; 2 Department of Pathology, University Hospital, School of Medicine, University of Crete, Heraklion, Greece; 3 Department of Surgical Oncology, University Hospital, School of Medicine, University of Crete, Heraklion, Greece; 4 Department of Obstetrics and Gynaecology, University Hospital, School of Medicine, University of Crete, Heraklion, Greece

## Abstract

Patient selection for PI3K-targeted solid cancer treatment was based on the *PIK3CA/PTEN* mutational status. However, it is increasingly clear that this is not a good predictor of the response of breast cancer cells to the anti-proliferative effect of PI3K inhibitors, indicating that isoform(s) other than p110α may modulate cancer cells sensitivity to PI3K inhibition. Surprisingly, we found that although no mutations in the p110δ subunit have been detected thus far in breast cancer, the expression of p110δ becomes gradually elevated during human breast cancer progression from grade I to grade III. Moreover, pharmacological inactivation of p110δ in mice abrogated the formation of tumours and the recruitment of macrophages to tumour sites and strongly affected the survival, proliferation and apoptosis of grafted tumour cells. Pharmacological inactivation of p110δ in mice with defective macrophages or in mice with normal macrophages but grafted with p110δ-lacking tumours suppressed only partly tumour growth, indicating a requisite role of p110δ in both macrophages and cancer cells in tumour progression. Adoptive transfer of δ^D910A/D910A^ macrophages into mice with defected macrophages suppressed tumour growth, eliminated the recruitment of macrophages to tumour sites and prevented metastasis compared with mice that received WT macrophages further establishing that inactivation of p110δ in macrophage prevents tumour progression. Our work provides the first in vivo evidence for a critical role of p110δ in cancer cells and macrophages during solid tumour growth and may pave the way for the use of p110δ inhibitors in breast cancer treatment.

## Introduction

The class IA subset of phosphoinositide-3 kinases (PI3Ks) are heterodimers made up of a regulatory subunit and a 110 kDa catalytic subunit (p110α, p110β or p110δ)^1–3^. The p110α isoform is a well-known oncoprotein since gain-of-function mutations in *PIK3CA* gene are present in a wide variety of human solid tumours^4–7^. The p110β isoform has been implicated in platelet biology, thrombosis^8^ and in certain cancers, especially in tumour cells lacking phosphatase and tensin homolog on chromosome 10  (PTEN)^9–13^. The gene encoding p110δ is rarely mutated in cancers^6,14–21^, and because of the preferential expression of p110δ in leukocytes this isoform has been mostly considered as a target in immunity and inflammation^22–24^. PI3K p110δ has been found to play also a seminal role in lymphoid and myeloid malignancies^25–29^ and a potent p110δ-selective inhibitor, Idelalisib, has been recently approved for the treatment of haematologic malignancies^30^. A promising role of the PI3K p110δ has also been suggested in cancers of non-haematopoietic origin^29^; however, the role of p110δ in breast cancer in vivo is poorly explored.

Deregulated PI3K signalling in breast cancer has frequently been attributed to gain-of-function mutations in *PIK3CA* gene, encoding the PI3K p110α and/or to loss-of-function mutations in the *PTEN* gene^6,31–35^. For this reason, the *PIK3CA*/*PTEN* mutational status was originally considered as a predictive molecular parameter of the sensitivity of cancer cells to PI3K inhibitors. However, recent studies have shown that there is no good correlation between the *PIK3CA* or *PTEN* mutational status and the response of breast cancer cells to the anti-proliferative effect of PI3K inhibitors^36–38^ indicating that unidentified mechanisms and/or isoform(s) other than p110α modulate the sensitivity of breast cancer cells to PI3K inhibition. An equally unexplained observation is that although *PTEN* somatic mutations are not very often in human breast cancers^39^, the deregulated PI3K activity in breast cancer cells is not controlled by wild-type (WT) PTEN.

Based on these observations, our work aimed to study a putative role of the PI3K p110δ in breast tumour progression providing new guidance for a potentially beneficial use of p110δ-selective inhibitors alone or in combination with inhibitors of other components of PI3K pathway. We report that pharmacological inactivation of p110δ blocks breast tumour growth by targeting cancer cells and macrophages. Our work also shows that the expression of p110δ is gradually increased during breast cancer progression and this correlates with a gradually decreased activity of PTEN, which overcomes and remains constantly high upon pharmacological inactivation of p110δ. These results strongly provide a rationale for considering the use of p110δ-selective inhibitors in breast cancer treatment and for the establishment of p110δ expression as a useful prognostic marker for the response of tumours expressing WT PTEN to p110δ inhibitors.

## Results

### Human breast cancer progression correlates with elevated PI3K p110δ expression levels

**Fig. 1:**
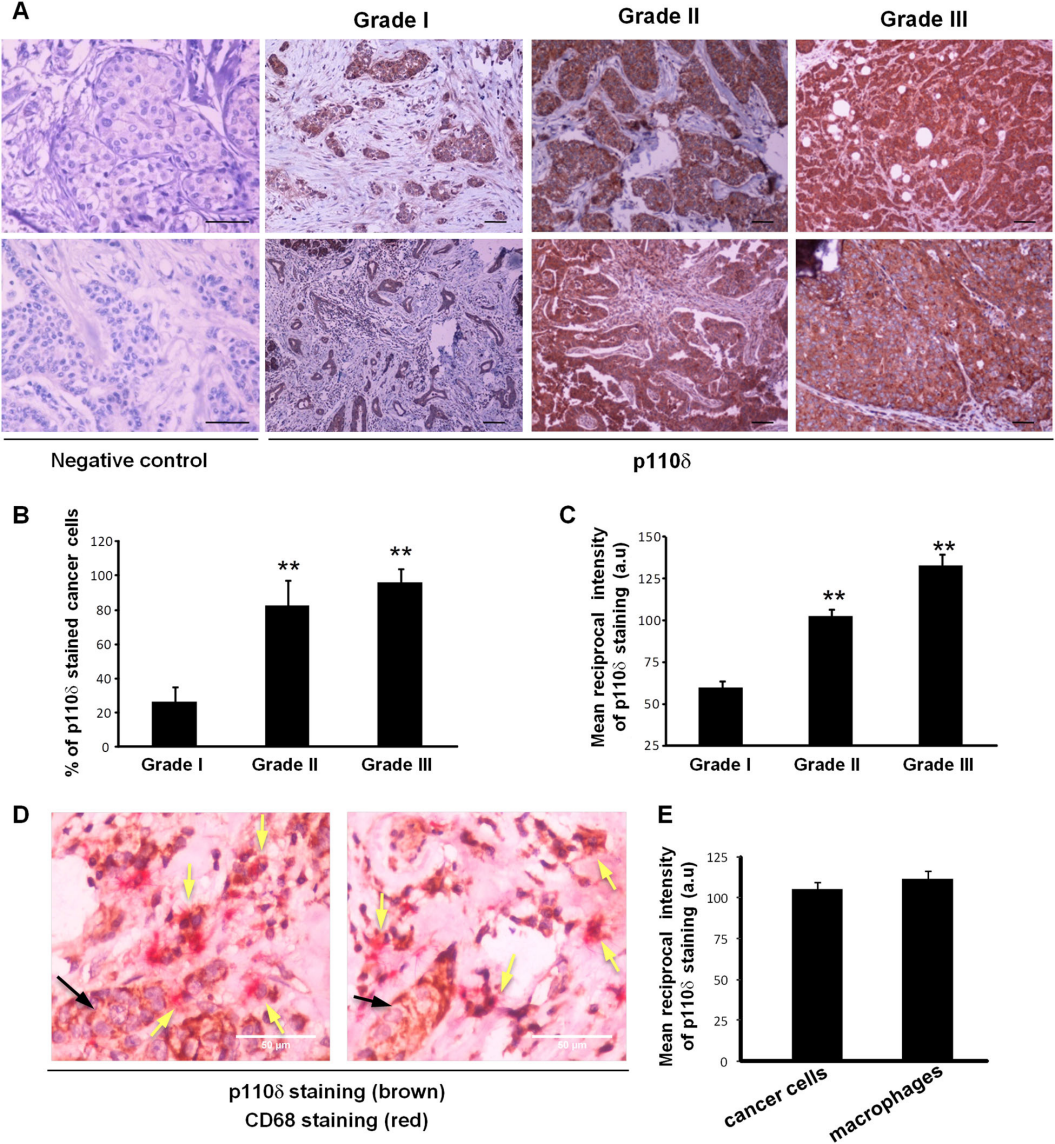
Impact of human breast tumour progression on p110δ expression levels. **a** Representative images of human breast cancer tissue sections (2 out of 20 for each grade) of grade I, grade II and grade III tumours stained with anti-p110δ antibody (brown) and haematoxylin (blue). In negative control sections, the anti-p110δ antibody was substituted by rabbit IgG. Scale bar = 100 μm. **b** Quantitation (means ± s.e.m) and comparison of the p110δ-positive cancer cells in tumours of grade I, grade II and grade III. **c** Quantitation (means ± s.e.m) of reciprocal intensity of the p110δ-stained cancer cells in tumours of grade I, grade II and grade III. **d** Double immunohistochemical staining of p110δ (brown) and CD68 (red) in two representative human breast cancer tissue sections of grade III tumours. Yellow arrows show macrophages double stained with anti-CD68 and anti-p110δ antibodies. Black arrows show p110δ staining in cancer cells in a representative tumour site. Scale bar = 50 μm. **e** Quantitation (means ± s.e.m) of reciprocal intensity of the p110δ-stained cancer cells and macrophages. Statistically significant differences are indicated by ** (*P* < 0.01), as determined by the Mann–Whitney test

Given that no somatic mutations of *PIK3CD* gene encoding the PI3K p110δ have been reported in breast cancer^6,14–17^, we sought to explore the p110δ protein expression during human breast cancer progression by immunohistochemistry in a collection of human breast carcinomas of grade I (*n* = 20), grade II (*n* = 20) and grade III (*n* = 20). p110δ was detected as cytoplasmic staining in all carcinomas and in some cases p110δ was additionally detected as focal staining and in deposits (Fig. 1a). However, p110δ staining positivity, quantified either as the percentage of p110δ-stained cancer cells (Fig. 1b) or as mean reciprocal intensity of p110δ staining in cancer cells (Fig. 1c), was much higher in grade II and III carcinomas compared with grade I carcinomas. We also assessed the expression of p110δ in macrophages in human breast tumours. Double immunostaining of human breast carcinomas of grade III for p110δ and CD68 revealed strong p110δ staining in macrophages (CD68+ cells) (Fig. 1d), which had an intensity similar with that measured in cancer cells (Fig. 1e). To confirm that the anti-p110δ antibody specifically recognizes the p110δ isoform, we tested it on sections from ovarian and cervical human cancers, which are known to express very low levels of p110δ but high levels of p110α and/or p110β^40^. Indeed, the anti-p110δ antibody detected very low p110δ immunoreactivity in those sections whereas strongly stained p110δ on breast cancer sections (Figure S1).

The collection of the human breast carcinomas used for the evaluation of p110δ levels was consisting of estrogen receptor (ER)- and/or progesterone receptor (PR)- positive, as well as ER-and/or PR-negative tumours whereas all tumours were human epidermal growth factor receptor 2 (HER2) negative. The results were similar in all samples regardless of ER or PR assignment, indicating that the p110δ staining does not correlate with the ER- or PR-positivity.

The above results indicate that the PI3K p110δ might play a functional role in human breast cancer.

### Oral administration of the IC87114 p110δ-selective inhibitor abrogates breast tumour growth in vivo and prevents the localization of macrophages into tumour sites

**Fig. 2:**
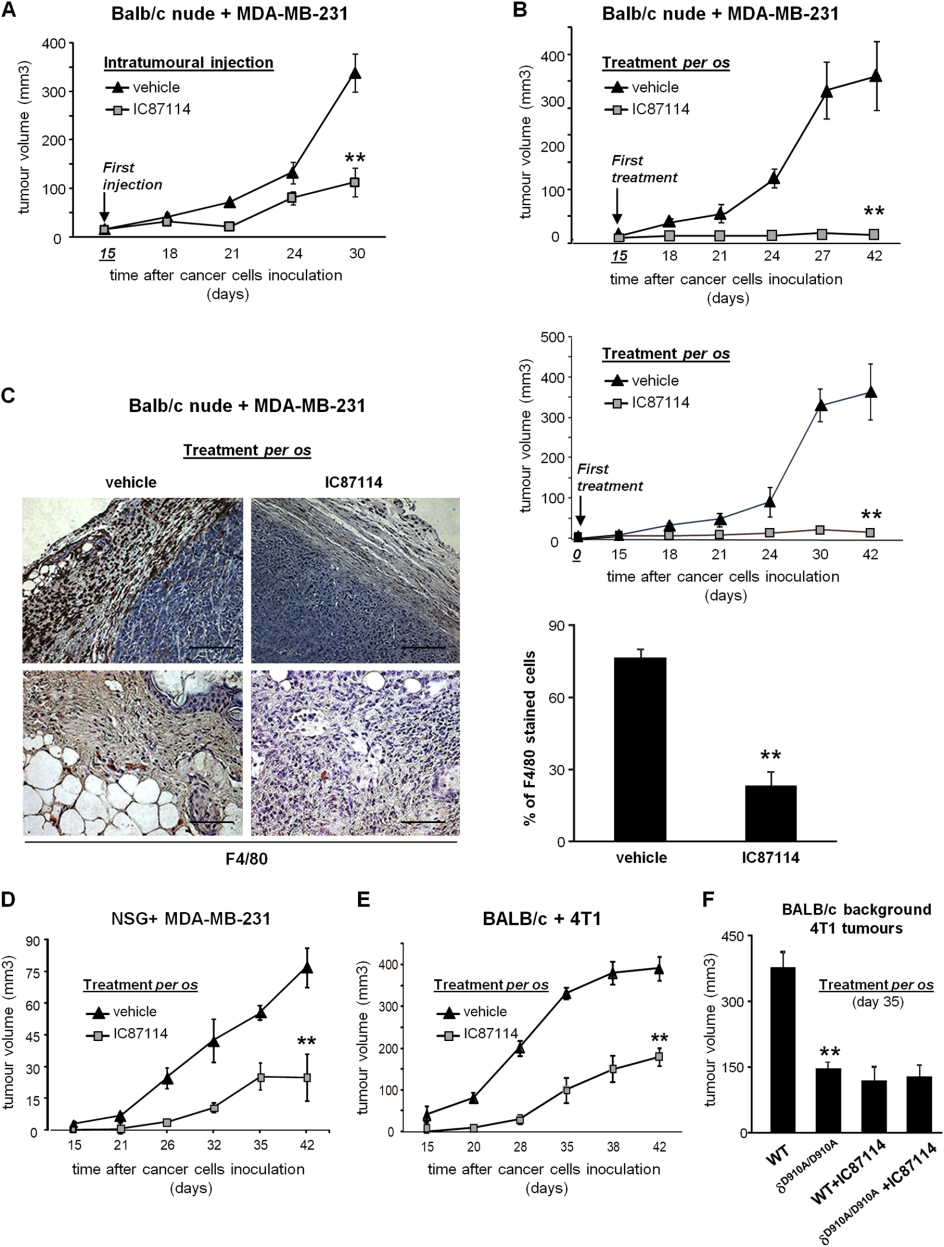
Impact of pharmacological inactivation of p110δ on breast tumour growth and on the recruitment of macrophages to tumour sites. **a** Growth of primary MDA-MB-231 tumours, inoculated in the breast fat pad of BALB/c nude mice, measured by digital callipers and expressed as tumour volume. Mice were treated intratumourally with vehicle or IC87114 (35 mg/kg). The drug was administered on day +15 and on every other day until day 30. *n* = 7 mice/group. **b** Growth of primary MDA-MB-231 tumours, inoculated in the breast fat pad of BALB/c nude mice, measured by digital callipers and expressed as tumour volume. Mice were treated once daily *per os* with vehicle or IC87114 (35 mg/kg) from day +15 (upper panel) or from day 0 (lower panel). *n* = 9 mice/group. **c** Immunohistochemical staining of macrophage-specific antigen F4/80 (brown) and haematoxylin (blue) in two representative sections of tumours excised from BALB/c nude mice that were treated *per os* with vehicle or IC87114 (35 mg/kg) (left panel). Scale bar = 50 μm. Comparison of F4/80-positive cells in tumours of IC87114-treated and vehicle-treated mice (right panel). **d** Growth of primary MDA-MB-231 tumours, inoculated in the breast fat pad of NOD *scid* gamma (NSG) mice, measured by digital callipers and expressed as tumour volume. Mice were treated once daily *per os* with vehicle or IC87114 (35 mg/kg) from day +15. *n* = 9 mice/group. **e** Growth of primary 4T1 tumours, inoculated in the breast fat pad of BALB/c mice, measured by digital callipers and expressed as tumour volume. Mice were treated once daily *per os* with vehicle or IC87114 (35 mg/kg) from day +15. *n* = 9 mice/group. **f** Growth of primary 4T1 tumours, inoculated in the breast fat pad of WT or δ^D910A/D910A^ mice on BALB/c background, measured by digital callipers and expressed as tumour volume. Mice were treated once daily *per os* with vehicle or IC87114 (35 mg/kg) from day +15. *n* = 10 mice/group. All graphs represent means ± s.e.m. Statistically significant differences are indicated by ** (*P* < 0.01), as determined by the Mann–Whitney test

To test the hypothesis that the PI3K p110δ has an essential role in tumour growth, we inoculated the MDA-MB-231 human breast cancer cell line into Balb/c nude mice to generate mammary tumours. MDA-MB-231 cells express high levels of p110δ comparable with those expressed in macrophages (Figure S2 and ref. ^40^). Intratumoural administration of IC87114, a small molecule inhibitor with selectivity for p110δ^41^, from day +15, when the tumour was palpable, significantly reduced tumour volume (Fig. 2a) suggesting that the inhibition of p110δ affects the proliferation of cancer cells and/or cells of the tumour microenvironment. However, tumours kept growing in smaller size (Fig. 2a). Surprisingly, when IC87114 was administered *per os* there was an almost complete lack of tumour growth (Fig. 2b) suggesting that p110δ needs to be additionally inactivated possibly in a sub-population of white blood cells, which when recruited to tumour sites counterbalance the effect of the intratumoural administration of IC87114 on tumour growth. The greater efficacy of oral treatment versus intratumoural injection of IC87114 seems not to depend on the timing of first administration. Indeed, similar blockade of tumour growth was achieved whether the inhibitor was administered *per os* from day +15 (Fig. 2b, upper panel), which was also the time of first intratumoural injection, or starting on the day of tumour cell engraftment, day 0 (Fig. 2b, lower panel).

Balb/c nude mice lack T cells but produce other subtypes of white blood cells. In recent years, tumour-associated macrophages (TAMs) have received great attention as key players in supporting tumour progression since they produce factors, which promote breast tumour growth and angiogenesis, suppress the anticancer immune responses and enhance the invasive potential of breast cancer cells^42–44^. To explore this possibility, we tested the impact of oral administration of IC87114 or vehicle on the recruitment of macrophages to tumour sites in MDA-MB-231-bearing Balb/c nude mice. Immunostaining of tumour samples for the macrophage-specific antigen F4/80 revealed that the abundance of macrophages, including those in the tumour stroma and tumour-infiltrating macrophages (Fig. 2c, left panel), was drastically reduced in mice receiving *per os* IC87114 (Fig. 2c, right panel). These data suggest that under *per os* treatment conditions, the inhibition of PI3K p110δ has a negative impact on the survival and/or recruitment of macrophages to tumour environment, which may additionally account for tumour regression.

To further assess the functional importance of the PI3K p110δ in both macrophages and cancer cells, we evaluated the impact of p110δ inhibition on tumour growth in two specific combinations of host mice and tumour cells: we used tumour cells expressing p110δ inoculated into mice with defective macrophages and tumour cells lacking p110δ growing up in mice with normal macrophages. In particular, we assessed (a) the growth of MDA-MB-231 tumours, which express p110δ in NOD *scid* gamma (NSG) mice that have defective macrophages and lack T cells (Fig. 2d and Supp Fig. 2) and (b) the growth of the syngeneic 4T1 tumours, which do not express p110δ in the Balb/c strain mice, which have normal macrophages and T cells (Fig. 2e and Supp Fig. 2). Oral administration of IC87114 either in MDA-MB-231 tumour-bearing NSG mice (Fig. 2d) or in 4T1 tumour-bearing Balb/c mice (Fig. 2e) reduced, but did not entirely block, tumour growth in both cases. Abrogation of tumour growth was only observed in *per os*-treated MDA-MB-231 tumour-bearing Balb/c nude mice, which, although lack T cells, have normal macrophages expressing p110δ and p110δ-expressing tumours (Fig. 2b).

We also assessed the growth of 4T1 tumours in δ^D910A/D910A^ mice in which the endogenous p110δ kinase has been genetically inactivated^45^. In parallel, we investigated the impact of IC87114 on tumour growth in WT and δ^D910A/D910A^ mice. Genetic inactivation of p110δ in host animals suppressed 4T1 tumour growth to a similar extend as pharmacological inactivation of p110δ in WT mice. Importantly, IC87114 did not further affect tumour growth in δ^D910A/D910A^ mice (Fig. 2f) confirming that IC87114 is an ‘on target’ p110δ inhibitor in vivo.

The above data suggest that the PI3K p110δ needs to be inactivated in both cancer cells and macrophages for an efficient blockade of tumour growth.

### Inactivation of the PI3K p110δ in macrophages is sufficient to confer tumour growth regression and to prevent metastasis

**Fig. 3:**
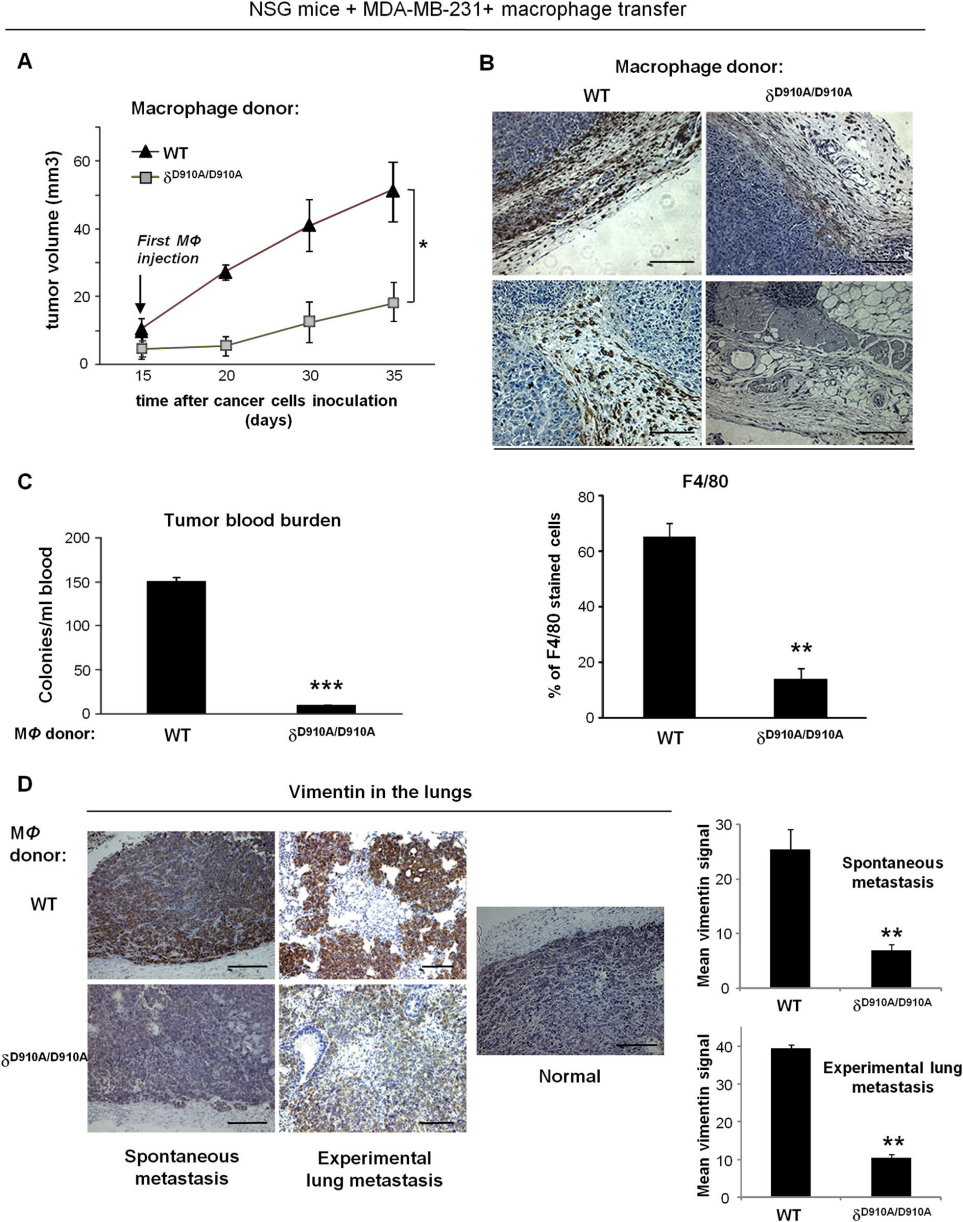
Inactivation of the PI3K p110δ in macrophages is sufficient to suppress tumour growth and metastasis. **a** Impact of adoptive transfer of δ^D910A/D910A^ macrophages into NSG mice on MDA-MB-231 primary tumour growth. *n* = 8 mice/group, * (*P* < 0.05) as determined by the Mann–Whitney test. **b** Impact of adoptive transfer of WT or δ^D910A/D910A^ macrophages into NSG mice on the recruitment of macrophages to tumour sites. Representative images of immunohistochemical staining with anti-F4/80 antibody (brown) and haematoxylin (blue) in two representative sections of tumours from NSG mice received WT or δ^D910A/D910A^ macrophages (upper panel). Scale bar = 50 μm. Comparison of F4/80-positive cells in tumours from NSG mice received WT or δ^D910A/D910A^ macrophages (lower panel). **c** Intravasation efficiency of cancer cells as determined by tumour cells blood burden at the end point of spontaneous metastasis in NSG mice, which received WT or δ^D910A/D910A^ macrophages (MΦ). *n* = 16, *** (*P* < 0.001), as determined by the Mann–Whitney test. **d** Invasion of cancer cells as determined by immunohistological staining of vimentin (brown) in the lungs of NSG mice, which received WT or δ^D910A/D910A^ macrophages during the spontaneous and experimental lung metastasis assay. Normal lung is also shown. Scale bar = 50 μm. Graphs represent quantification of the vimentin signal in tissue samples of the respective lungs (right panels). All graphs represent means ± s.e.m. Statistically significant differences are indicated by ** (*P* < 0.01), as determined by the Mann–Whitney test

To further confirm the functional impact of the PI3K p110δ inactivation in macrophages on tumour progression, we carried out adoptive macrophage transfer experiments in MDA-MB-231 tumour-bearing NSG mice. When δ^D910A/D910A^ macrophages, which express genetically inactivated p110δ^45^ were transferred into NSG mice, the tumour burden was significantly reduced compared with that observed in mice that received WT macrophages (Fig. 3a). In addition, immunostaining of tumour samples with the macrophage-specific antigen F4/80 (Fig. 3b, upper panel) showed that the abundance of macrophages into tumour sites was significantly reduced in mice receiving δ^D910A/D910A^ macrophages compared with mice receiving WT macrophages (Fig. 3b, lower panel).

We then assessed the role of the PI3K p110δ in macrophages in controlling cancer cells metastasis by determining the tumour cell blood burden and the expression of vimentin in the lungs. The tumour cell number in the blood collected from the right atrium of the heart before filtration by the lungs, is a direct evaluation of intravasation and therefore of efficiency of metastasis^46^, whereas elevated expression of vimentin is correlated with lung invasion of cancer cells^47,48^. To evaluate the impact of p110δ inactivation in macrophages on early and late steps of metastasis, we used MDA-MB-231 tumour-bearing NSG mice for spontaneous metastasis and NSG mice in which experimental lung metastasis assay^46^ was performed followed by the adoptive transfer of macrophages. Under conditions of adoptive transfer of δ^D910A/D910A^ macrophages in MDA-MB-231 tumour-bearing NSG mice, the tumour blood burden was significantly reduced (Fig. 3c) indicating that the spontaneous intravasation and therefore metastasis is prevented when p110δ in macrophages is inactive. Similarly, transfer of δ^D910A/D910A^ macrophages either into NSG mice bearing MDA-MB-231 tumours or into mice with experimental lung metastasis led to reduced expression of vimentin in the lungs (Fig. 3d) reflecting decreased invasion of cancer cells.

Together, these data indicate that targeting of PI3K p110δ in macrophages is sufficient to prevent the localization of macrophages into tumour sites and consequently to suppress tumour growth and metastasis.

### Pharmacological inactivation of the PI3K p110δ affects the survival, proliferation and apoptosis of tumour cells

**Fig. 4:**
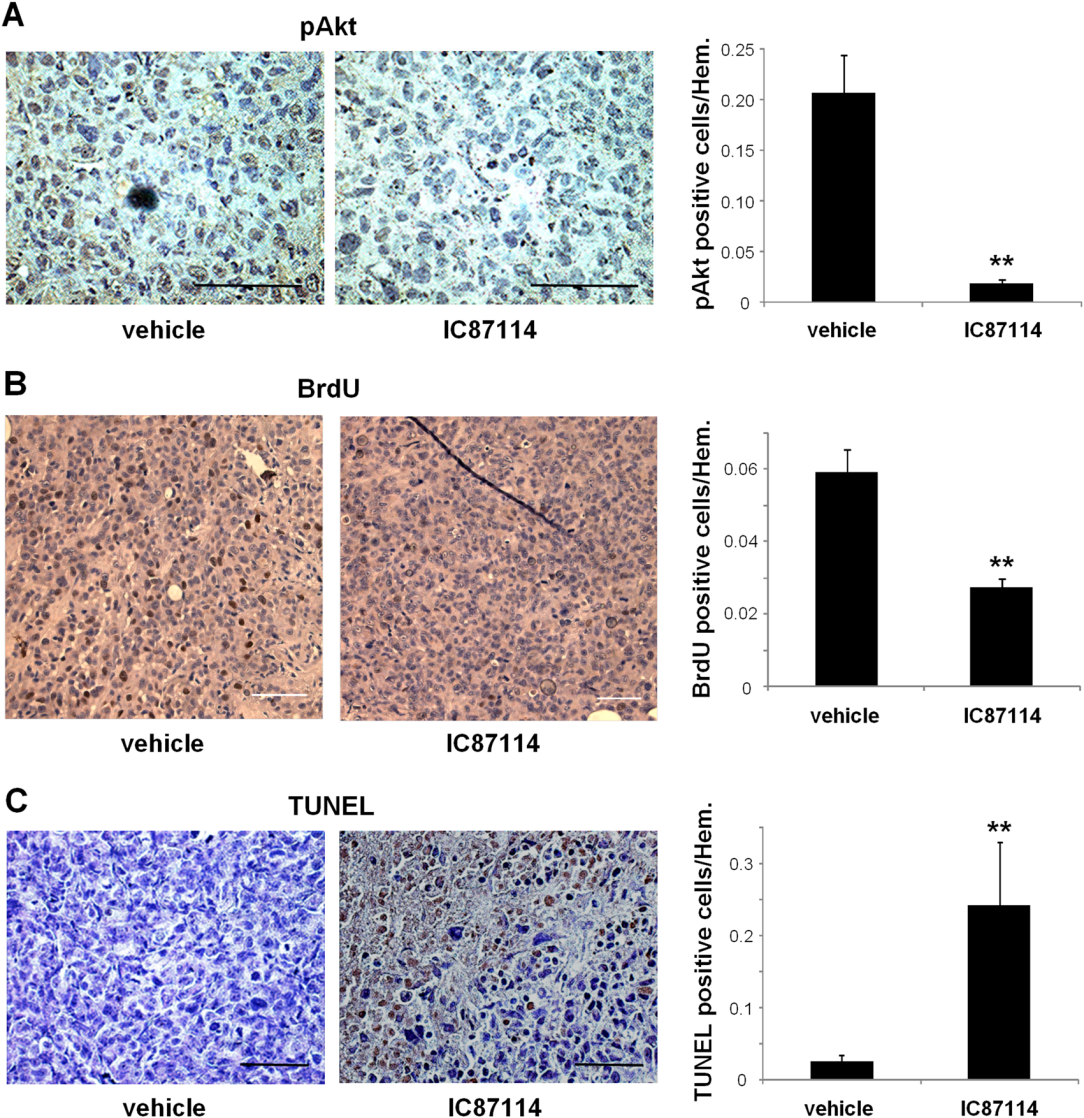
Efficacy of pharmacological inactivation of the PI3K p110δ against tumour cells. **a** MDA-MB-231 primary tumours harvested from IC87114 (35 mg/kg)-treated and vehicle-treated BALB/c nude mice were subjected to immunohistochemical analysis using anti p-Akt antibody (brown) and haematoxylin (blue) (left panels). Scale bar = 25 μm. Comparison of p-Akt-positive cells in tumours of IC87114-treated and vehicle-treated mice (right panel). **b** Cell proliferation in tumours of IC87114-treated and vehicle-treated BALB/c nude mice was determined by BrdU incorporation (brown spots) (left panels). Scale bar = 50 μm. Comparison of BrdU-positive cells in tumours of IC87114-treated and vehicle-treated mice (right panel). **c** Apoptosis in tumours of IC87114-treated and vehicle-treated BALB/c nude mice was determined by TUNEL assay (brown spots) (left panels). Scale bar = 50 μm. Comparison of TUNEL-positive cells in tumours of IC87114-treated and vehicle-treated mice (right panel). All graphs represent means ± s.e.m. Statistically significant differences are indicated by ** (*P* < 0.01), as determined by the Mann–Whitney test

To assess the direct effect on tumour cells, we investigated whether the treatment of BALB/c nude mice with the p110δ inhibitor *per os* affects the survival, proliferation rate and apoptosis of tumour cells and can therefore account for the reduced tumour growth. The phosphorylated Akt (Fig. 4a), as well as the 5-bromo-2′-deoxyuridine (BrdU)-positive cells (Fig. 4b) were significantly reduced in tumour specimens from mice treated with IC87114 compared with vehicle, indicating reduced survival and proliferative rate of tumours upon p110δ inhibition. The number of terminal deoxynucleotidyl transferase-mediated dUTP nick end labelling (TUNEL)-positive cells was also significantly increased in IC87114-treated mice (Fig. 4c), suggesting induced apoptosis in tumour cells.

Taken together, the above results show that pharmacological inhibition of the PI3K p110δ prevents tumour progression by directly affecting both cancer cells and macrophages.

### Elevated p110δ expression levels correlate with reduced PTEN activity in late tumour stages

**Fig. 5:**
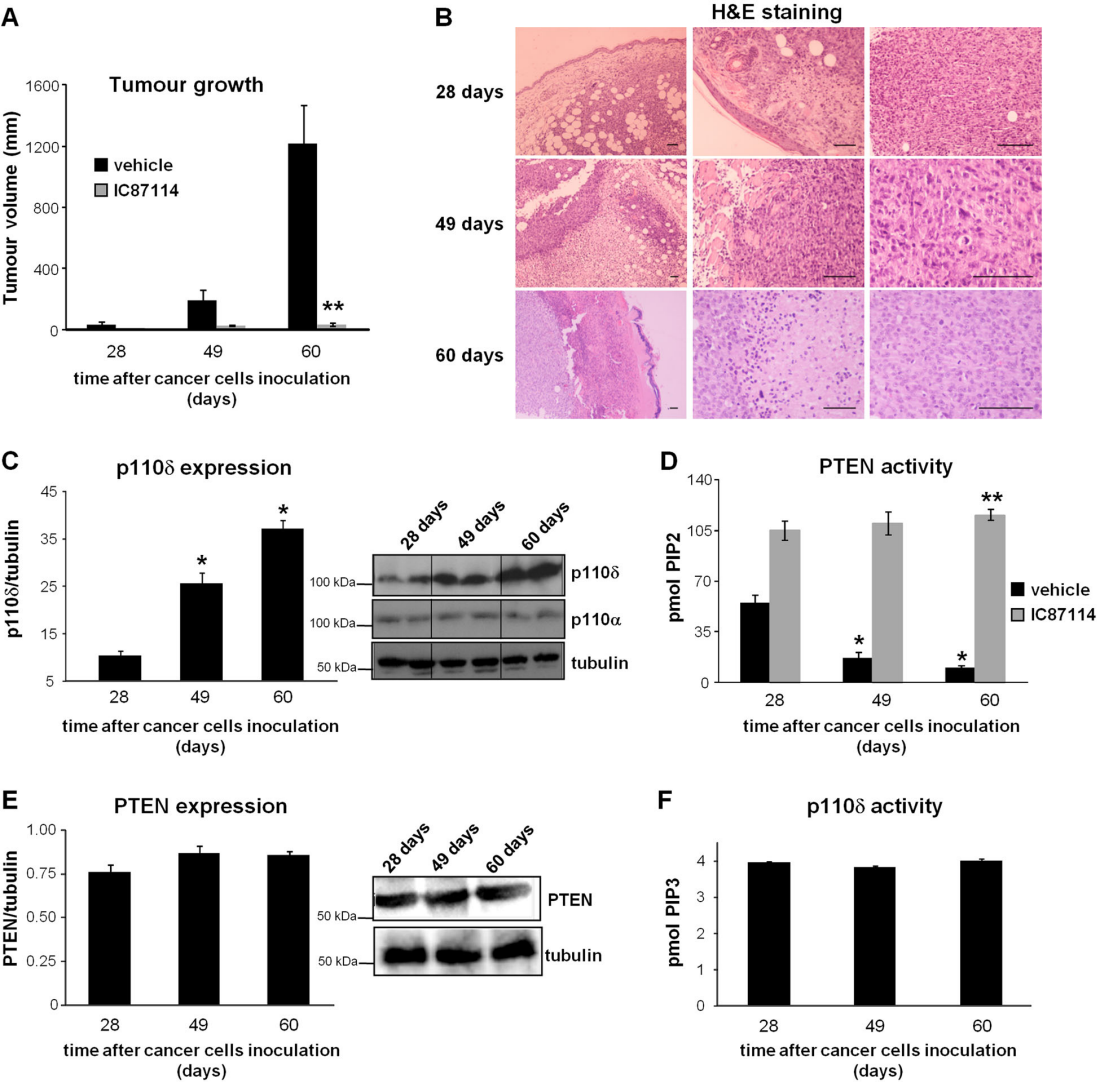
Efficacy of the PI3K p110δ inactivation on PTEN activity in tumour cells. **a** MDA-MB-231 primary tumour burden in vehicle-treated and IC87114 (35 mg/kg)-treated BALB/c nude mice at the indicated times. *n* = 6–10 mice/group. **b** H&E staining of the tumours excised from vehicle-treated mice of the experiment described in (**a**). Scale bar = 50 μm. **c** p110δ and p110α expression levels in tumour cells that were isolated from harvested tumours of vehicle-treated mice. **d** PTEN activity in tumour cells isolated from harvested tumours of vehicle-treated and IC87114-treated mice. **e** PTEN expression levels in tumour cells isolated from harvested tumours of vehicle-treated mice. **f** p110δ activity in tumour cells isolated from harvested tumours of vehicle-treated mice. All graphs represent means ± s.e.m. Statistically significant differences are indicated by * (*P* < 0.05) or ** (*P* < 0.01), as determined by the Mann–Whitney test

The finding described above showing that the expression of the PI3K p110δ becomes elevated during human breast cancer progression (Fig. 1) indicates that high p110δ expression may associate with enhanced survival of cancer cells. In an attempt to identify the mechanism that could explain how high p110δ expression in advanced tumour stages associates with the survival of cancer cells, we assessed a potential correlation between p110δ expression levels and PTEN activity during tumour growth, based on previous findings of ours showing that the two correlate in breast cancer cell lines^40,49^. Balb/c nude mice receiving vehicle alone, were left to develop MDA-MB-231 tumours for different time periods (Fig. 5a, black bars) in an attempt to mimic breast tumour progression from early to late stages; the excised tumours were first examined by histology (Fig. 5b). Cancer cells characterized by large nucleus, nucleoli and undergoing mitosis and hair follicles with infiltrating cancer cells in otherwise healthy epidermis were detected in tissue specimens from mice that carried the tumours for up to 28 days (Fig. 5b). Healthy epidermal and stromal cells were also found in these tumours (Fig. 5b). Cancer cells under mitosis, muscle infiltration of the tumour and large necrotic areas, which reflect the development of invasive cancer and tumour aggressiveness, were present in the excised tissues when tumours were left to grow for up to 49 days (Fig. 5b). Allowing further tumour growth for up to 60 days showed characteristics indicating that tumours outgrew their blood supply such as necrotic areas in the tumour and necrotic epidermis. Mitotic cancer cells were also still observed (Fig. 5b).

We then assessed the expression and activity of p110δ and PTEN in these tumours. The expression levels of p110δ protein gradually increased during tumour growth in mice (Fig. 5c), which is in accordance with that we found in human tumour samples (Fig. 1), whereas the expression levels of p110α, used as control, were unaffected (Fig. 5c). In contrast, the activity of PTEN gradually decreased (Fig. 5d, black bars). The expression levels of PTEN protein and the enzymatic activity of p110δ remained constant during tumour growth (Figs. 5e, f respectively).

We additionally investigated the impact of p110δ inhibition on PTEN activity in tumour cells. Oral treatment of parallel groups of mice with the p110δ-selective inhibitor, IC87114, which prevented tumour growth (Fig. 5a), significantly increased PTEN activity in tumour cells (Fig. 5d), indicating that a mechanism by which the p110δ inhibition suppresses the survival of tumour cells is via the increased PTEN activity. The activity of p110δ was determined in the same tumour cells that PTEN activity was evaluated and found to be undetectable confirming the functional effectiveness of the inhibitor (data not shown).

Taken together, these data strongly suggest that the enhanced growth of cancer cells during breast tumour progression is at least in part a result of a gradually reduced activity of PTEN, which correlates with a parallel increase in p110δ expression levels.

## Discussion

Our work shows that the PI3K p110δ plays a prominent role in breast cancer progression and that pharmacological inactivation of p110δ in mouse experimental models prevents breast tumour growth and metastasis by targeting both cancer cells and macrophages.

The predominant expression of p110δ in white blood cells^50,51^ has shifted the interest in the biology of the PI3K p110δ pathway toward immune disorders and haematologic cancers^52^; p110δ-selective inhibitors have been studied in multiple haematologic malignancies^25,26^. The antitumour activity of the initially developed CAL-101 inhibitor (GS-1101 or Idelalisib)^26,53^ is mainly attributed to disruption of the survival and adhesion signals conferred by supporting cells of the B-CLL microenvironment^25,52,54,55^. It is of note that, the targeting of PI3K and its downstream kinases by single-agent inhibitors has limited efficacy with the exception of the PI3K p110δ-selective inhibitors, which target the tumour stroma and have shown a remarkable clinical activity in CLL^52^.

Our present results clearly show that the effectiveness of the IC87114 p110δ-selective inhibitor in preventing breast tumour growth and metastasis is not only a result of its efficacy to inhibit the survival and proliferation of cancer cells but also derives from the modulation of macrophage recruitment to tumour sites. We show that the localization of macrophages in tumour stroma was drastically decreased by IC87114 treatment, reflecting a similar mechanism with the one observed with the p110δ inhibitors in CLL.

The reduced abundance of TAMs in the tumour mass most likely results from an effect of p110δ inactivation on survival of TAMs and/or on the recruitment of macrophages to tumour sites. The fact that the localization of macrophages into tumour sites was eliminated in NSG mice that received δ^D910A/D910A^ macrophages suggests that p110δ inactivation mainly affects the recruitment of macrophages to tumour sites. Inactivation of PI3K p110δ has been previously shown to block chemotaxis of macrophages^49^ and to affect their polarization^56^ explaining the reduced recruitment of macrophages to tumour environment upon p110δ blockade. It is known that the process that macrophages promote the invasion and intravasation of breast cancer cells occurs via association of tumour cells with perivascular TAMs^57–61^. The adoptive transfer of δ^D910A/D910A^ macrophages in NSG mice prevented the recruitment of macrophages to tumour environment and suppressed intravasation and metastasis of tumour cells indicating a critical role of PI3K p110δ in macrophages in controlling breast cancer metastasis.

It has been recently shown that inhibition of the PI3K p110γ decreases tumour growth by shifting the transcription program in macrophages toward enhanced adaptive immunity, including increased recruitment and cytotoxicity of T cells^62,63^, but without affecting *per se* the accumulation of macrophages in tumours^62^. This seems to be a distinct mechanism from the emerging from the present study. In fact, our results clearly show that inactivation of the PI3K p110δ significantly reduces the abundance of macrophages in the primary tumour site and almost completely blocks tumour formation in Balb/c nude mice, which lack T cells. It has also been proposed that inhibition of the PI3K p110δ decreases tumour growth by reducing the immune-suppressive function of regulatory T cells (Tregs) thus enhancing an antitumour cytotoxic T-cell response^64^. The effect of p110δ inhibition on the abundance of macrophages to tumour sites that we show here might include the effect of p110δ inhibition on Treg function because TAMs are known to mediate their immunoregulatory function through various mechanisms including the CCL22-mediated recruitment of Treg cells^65^. However, the reduced localization of macrophages into tumour sites seems to impact on tumour growth by triggering additional mechanisms since inactivation of p110δ blocks tumour growth of p110δ-expressing tumours even in the absence of T cells (such as in Balb/c nude mice (Fig. 2b)). The inactivation of p110δ in MDA-MB-231-bearing tumour NSG mice (which have defective macrophages and lack T cells while their tumours express p110δ) or in 4T1-bearing tumour Balb/c mice (which have normal macrophages and normal T cells but their 4T1 tumours do not express p110δ) suppresses but is not able to entirely block tumour formation (Figs. 2d, e) pointing at an indispensable role of p110δ inactivation in both macrophages and cancer cells for breast tumour growth blockade.

Based on the remarkable clinical impact of the PI3K p110δ inhibitors in blood cancers, especially in some human B-cell malignancies^29,66^, one can postulate that B cells may mediate, at least in part, the effects we observe here. This, however, is not supported by what is known about the role of B cells in solid tumours, with conflicting reports as to whether B-cell presence in solid tumours is a marker of good or bad prognosis^67^. If an association of B cells with good outcomes is the case, then a possible decrease of infiltrating B cells by a p110δ inhibitor would conflict with the favourable outcome we present here. On the other hand, a tumour-promoting role of CD20+ B cells have been proposed in early breast cancer^68^; however, this does not seem to play a role in the results we show here since tumour growth was prevented by pharmacological blockade of p110δ even in pre-established tumours (Fig. 2b).

In many cancers, the increased presence of TAMs drives tumour progression and correlates with poor prognosis by promoting cancer cells proliferation, angiogenesis, immunosuppression, invasiveness and metastasis^42–44^. In breast cancer clinical studies, macrophages have been found to constitute 50% of the cell mass and to correlate with poor prognosis in 80% of the cases^58,69,70^. Furthermore, TAMs are known to influence the response to cancer therapies^42,71,72^. Therefore, a wealth of evidence suggests that combination approaches targeting both cancer cells and TAMs may be clinically beneficial^73,74^. Our findings show that pharmacological inactivation of the PI3K p110δ targets both the growth of tumour cells and the recruitment of macrophages to tumour sites leading to a remarkable suppression of breast tumour growth and metastasis.

In recent years, the *PIK3CA*/*PTEN* mutational status was considered as a molecular criterion for patient selection. However, accumulating unexplained observations have indicated that this parameter may not be as straightforward as originally hoped for. For example, breast cancer cells were found to be sensitive to growth inhibition by PI3K inhibitors without harbouring mutations in the *PTEN* or *PIK3CA* genes^37^; in addition, breast cancer cells with PTEN deficiency were found to be resistant to PI3K inhibitors^38^. Our findings show that although no mutations in p110δ have been detected thus far in breast cancer, the expression levels of p110δ are gradually increased during human breast cancer progression from grade I to grade III indicating that PI3K p110δ might play a prominent role in human breast cancer. In support of this, our data show that the gradually increased p110δ expression correlates with a gradually reduced PTEN activity, which is recovered and becomes elevated upon treatment with the p110δ inhibitor. PTEN is under the negative control of PI3K p110δ activity^49,75^ and we have also previously shown that the expression levels of the PI3K p110δ in breast cancer cell lines are associated with the activity of WT PTEN^40,49^. Given that *PTEN* somatic mutations are not very often in human breast cancers^39^, the expression levels of p110δ may represent a useful straightforward marker, which can predict an effective response of those tumours expressing WT PTEN to therapy with p110δ inhibitors.

In conclusion, our data uncover a major role of the PI3K p110δ in breast tumour progression and demonstrate that pharmacological inactivation of this isoform prevents tumour growth and metastasis by concomitantly attaching the two major therapeutic targets, named cancer cells and macrophages. Our data support and add to the emerging rationale for targeting PI3K in the tumour stroma and strongly argue in favour of considering the use of p110δ inhibitors in future clinical trials for breast cancer treatment.

## Materials and methods

### Chemicals

The PI3K p110δ inhibitor IC87114 was purchased from Chemietek and re-suspended in 30% PEG-400, 0.5% Tween-80 and 5% propylene glycol for experiments in mice.

### Cell culture

The MDA-MB-231 human breast cancer cell line was a gift from Bart Vanhaesebroeck (Ludwig Institute for Cancer Research, London, United Kingdom). Cells were cultured in Dulbecco’s modified Eagle’s medium (Invitrogen, Life Technologies) supplemented with 10% foetal calf serum and 1% penicillin–streptomycin at 37 °C with 5% CO_2_. Cells were regularly tested for mycoplasma infection. Prior to injection into mice, cells were cultured to 70% confluence, counted and re-suspended in sterile PBS.

### Mice

All mice were kept in a pathogen-free animal facility at the Institute of Molecular Biology and Biotechnology in Heraklion, Crete. All procedures were approved by the Research Animal Care Committee of Medical School, University of Crete and by the Veterinary Department of Heraklion Prefecture (protocol no. 2182), according to national and EU legislation. Female BALB/c nude mice were obtained from Charles River Laboratories. Female NOD.*Cg-Prkdc*^*scid*^ *Il2rg*^*tm1Wjl*^ (NOD *scid* gamma or NSG) mice were obtained from The Jackson Laboratory. δ^D910A/D910A^ mice were kindly provided by Professor Bart Vanhaesebroeck (UCL).

The choice of group sample size was based on calculations using the NC3Rs recommended Resource Equation method, pilot experiments and prior knowledge of the variability of breast tumours in BALB/c nude, NSG and BALB/c mice. Mice used in all experiments were female, age-matched (2–3 months of age) and in otherwise excellent physical health. The allocation of animals to experimental groups was performed by simple random sampling at the beginning of each experiment. All mice received injections of tumour cells and then equal number of mice was selected at random for each treatment arm. Each experiment was performed using a minimum of six mice per group (the exact number of mice is indicated in each figure legend) and was repeated at least three times with independent groups of animals to assess reproducibility.

### In vivo studies of tumour growth in xenograft and syngeneic models

Female BALB/c nude or NSG mice were inoculated subcutaneously into the right breast fat pad on day 0 with 10^6^ MDA-MB-231 cells in 100 μl phosphate-buffered saline (PBS), whereas BALB/c mice were inoculated with 4T1 cells. Mice were then randomly assigned into the control group receiving vehicle alone or into the treatment group receiving 35 mg/kg IC87114 by oral gavages once daily starting from day 0 or from day +15. When this is indicated, IC87114 was instead administered by intratumoural injection starting on day +15 when the tumour was palpable. Tumour growth was monitored by measurement of the longest perpendicular tumour diameters using a digital calliper every 3–6 days. The tumour volume (V) was calculated^76^ as V (mm^3^) = length (mm) × width (mm)^2^ × 0.5. At the end of the study, animals were euthanized and primary tumours and lungs were removed for haematoxylin and eosin (H&E) staining, immunohistochemistry or determination of PTEN and PI3K p110δ activity and expression levels as described below.

### Adoptive macrophage transfer, spontaneous and experimental metastasis assay

WT mice, or mice in which p110δ alleles are replaced by a kinase-dead version of p110δ (called δ^D910A/D910A^), mutated in the ATP binding site^45^, served as macrophage donor mice. WT and δ^D910A/D910A^ mice received intraperitoneal injections of 5 ml of 3% Brewer thioglycollate medium and 1 week later were euthanized and peritoneal macrophages were collected as previously described^77^. MDA-MB-231 cells were inoculated subcutaneously into the right mammary fat pad of recipient NSG mice on day 0 as described above and then randomly assigned into the control group receiving vehicle alone or into the treatment groups receiving macrophages from WT or δ^D910A/D910A^ mice. On day +15, 5 × 10^5^ macrophages from WT or δ^D910A/D910A^ mice were intravenously administered into NSG mice. The adoptive macrophage transfer was cycled every 48 h for 15 days. Tumour growth was monitored by measurement of the tumour diameters using a digital calliper every 5 days and the tumour volume (V) was calculated as described above. At the end of the study, mice were anaesthetised and tumour cell blood burden was determined as described below, followed by sacrifice of the mice and removal of the primary tumour and the lungs for immunohistochemistry as described below. For the experimental lung metastasis assay, 10^6^ MDA-MB-231 cells were injected into the lateral tail vein of NSG mice, followed 14 days later by intravenous administration of 5 × 10^5^ macrophages from WT or δ^D910A/D910A^ mice. The adoptive macrophage transfer was cycled every 48 h for 15 days. At the end of study, mice were anaesthetised before sacrifice and removal of lungs for immunohistochemistry as described below.

### Tumour cell blood burden

The tumour cell blood burden was measured as previously described^46^. Briefly, mice were anaesthetised and blood was collected from the right atrium by a syringe coated with heparin via heart puncture with a 25-gauge needle. The blood was plated into culture dishes in which tumour cells were selectively grown by using Geneticin. Tumour cell clones in the dish were counted and the tumour blood burden was calculated as the number of colonies in the dish divided by the volume of the blood taken.

### Immunohistochemistry

Paraffin-embedded and formalin-fixed tissues from mice were handled as previously described^78^. Briefly, tissue sections were deparaffinized and dehydrated through graded ethanol series. Sections were treated with 3% hydrogen peroxide for 30 min at 20 °C (to inactivate endogenous peroxidase activity) and rinsed in PBS. Epitope unmasking was performed by digestion with 0.2% trypsin for 10 min at room temperature (RT) (in the case of anti-pAkt antibody) or by heating for 40 min in citrate buffer (pH6.0) (in the case of anti-vimentin). The tissue sections were first blocked for nonspecific binding with goat serum (when pAkt antibody was used) or 1% bovine serum albumin for 1 h at RT (for the other antibodies) and then incubated with the primary antibodies overnight at 4 °C (1:50 for pAkt (Cell Signalling #4060), 1/50 for F4/80 (Bio-Rad #MCA497GA) and 1/200 for vimentin (Thermo Scientific #RM-9120)). The tissue sections were then incubated with an anti-rat (for F4/80) or an anti-rabbit horseradish peroxidase (for pAkt and vimentin) with DAB (3,3'-diaminobenzidine) and H_2_O_2_, counterstained with haematoxylin and mounted with Vectashield mounting medium (Vector Labs). The counting of pAkt-positive and F4/80-positive cells was performed using ImageJ software (NIH). Vimentin was quantified as the average pixel intensity per field of view using Scion Image freeware (Scion Corp., Frederick, MD, USA)^79^. Values are presented as means ± s.e.m of stained cells counted from 5 to 8 fields/section (randomly selected) and from 3 sections/determination. The immunohistochemisty experiments were performed using tissues from at least three repeat experiments. The results were similar among different experiments. For human cancer tissues, immunohistochemistry was performed on 4 μm thick sections of formalin-fixed, paraffin-embedded archival breast cancer tissues of the Pathology Department (University Hospital, Heraklion, Greece). Following deparaffinization and rehydration, the tissue sections where pre-treated by microwaving in EDTA solution (pH 8.0) for 15 min at 500 W followed by cooling at RT for 20 min. The anti-p110δ antibody (Abcam #ab200372) was incubated for 1 h at RT. The DAKO REAL EnVision Detection System K5007 was used according to the manufacturer’s instructions for visualization with DAB chromogen. For double staining, the anti-CD68 antibody (Thermo Scientific #MA5-13324) was subsequently incubated for 1 h at RT at 1/200 dilution and Envision G2 System was used according to the manufacturer’s instructions for visualization with alkaline–phosphatase (Permanent Red). Counterstaining was performed using Meyer’s haematoxylin. Immunohistochemical results were first evaluated in a semiquantitative manner and scored according to the percentages of positive stained cells (1+: 5–25% of cells stained, 2+: 25–50% of cells stained, 3+: 50–100% of cells stained). Cytoplasmic and focal staining was considered positive. The p110δ expression profile was concordant in all cases of each grade analyzed (20 individual cases of grade I, 20 individual cases of grade II and 20 individual cases of grade III).The counting of p110δ-positive cancer cells and the measurement of reciprocal intensity^80^ of the p110δ staining were performed using ImageJ software (NIH). Values are presented as means ± s.e.m. of the % of p110δ-stained cancer cells versus haematoxylin-stained cells or as means ± s.e.m. of the reciprocal intensity of p110δ staining in cancer cells or macrophages of randomly selected 6–12 fields/image and 3 images/patient for all patients.

### BrdU incorporation

Mice were injected intraperitoneally with 100 mg/kg bodyweight of BrdU (Calbiochem) 2 h before euthanasia and BrdU-positive tumour cells were detected using a BrdU staining kit (Millipore #2760) according to the manufacturer’s instructions. The counting of BrdU-positive cells was performed using ImageJ software (NIH). Values are presented as means ± s.e.m. of BrdU-positive/haematoxylin-stained cells counted from 5 to 8 fields/section (randomly selected) and 3 sections/measurement. The BrdU incorporation procedure was performed on tissues obtained from at least three separate experimental groups of animals for each treatment condition. The results were similar among the different experimental repeats.

### TUNEL assay

The detection of apoptosis was performed using the DeadEnd colorimetric TUNEL system (Promega #G7130), according to the manufacturer’s instructions. The counting of TUNEL-positive cells was performed using ImageJ software (NIH). Values are presented as means ± s.e.m. of TUNEL-positive/haematoxylin-stained cells counted from 5 to 8 fields/section (randomly selected) and 3 sections/measurement. The TUNEL procedure was performed on tissues obtained from at least three separate experimental groups of animals for each treatment condition. The results were similar among different experiments.

### Immunoprecipitation (IP) and Western blot (WB)

Cells were isolated from tumours using an enzyme mixture consisting of 0.05 mg/mL Collagenase I, 0.05 mg/mL Collagenase IV and 0.01 mg/mL DNase I in HBSS (Hank's Balanced Salt Solution) followed by isolation of cancer cells using the Τumor Cell Isolation kit (Cell Biolabs). Cells were lysed in lysis buffer containing 150 mM NaCl, 1,5 mM MgCl_2,_ 1 mM EGTA, 10% glycerol, 100 mM NaF, 25 mM glycerolphosphate, 1% IPEGAL (octylphenoxypolyethoxyethanol), 1 mM DTT (dithiothreitol), 10 mM Na-pyrophosphate, 1 mM PMSF (phenylmethylsulfonyl fluoride), 10 μg/ml aprotinin, 10 mM Na_4_ VO_3_ and 50 mM Hepes, pH 7.4 (in the case of the PTEN lipid phosphatase activity assay) or in lysis buffer containing 20 mM Tris-HCl, pH 7.4, 137 mM NaCl, 1 mM CaCl_2_, 1 mM MgCl_2_, 1 mM sodium orthovanadate, 1% NP-40 and 1 mM PMSF (in the case of the p110δ activity assay), followed by clearing of the lysates by centrifugation in a cooled microcentrifuge. Supernatants were directly immunoprecipitated at 4 °C overnight using an anti-PTEN (Santa Cruz Biotechnology #A2B1 or #sc-6818) or anti-p110δ (Abcam #ab1678) antibody respectively. Immune complexes were collected with 50% slurry of protein A-Sepharose after incubation for 2–3 h at 4° and washed according to the manufacturer’s instructions (Echelon Biosciences) provided the ELISA kits. For analysis of total cell lysates by western blotting, 50–70 μg of cell extract (of the same samples used for the activity assay) was loaded per lane on an sodium dodecyl sulphate–polyacrylamide gel electrophoresis gel and transferred onto PVDF (Polyvinylidene Difluoride) membranes. The blots were probed with the indicated antibodies (anti-PTEN (Santa Cruz Biotechnology #sc-9145) or anti-p110δ (Santa Cruz Biotechnology #sc-7176)), followed by detection using enhanced chemiluminescence (GE Healthcare).

### PTEN lipid phosphatase activity assay

PTEN was immunoprecipitated from cell lysates derived from tumour cells isolated from each tumour-bearing mouse from each experimental group and its lipid phosphatase activity measured by ELISA  (Enzyme-linked Immunosorbent Assay), according to the manufacturer’s instructions (Echelon Biosciences). The PI(4,5)P2 produced was determined by comparison with a standard curve consisting of PI(4,5)P2 standards bound to the ELISA plate. Different amounts of proteins of cell lysates, from which PTEN was immunoprecipitated, were tested in pilot experiments to ensure that the appropriate amount of enzyme were used so that the produced PI(4,5)P2 was in the range of the respective standard curve.

### PI3K p110δ lipid kinase activity assay

p110δ was immunoprecipitated from cell lysates derived from tumour cells isolated from each tumour-bearing mouse from each experimental group and its lipid kinase activity measured by ELISA, according to the manufacturer’s (Echelon Biosciences) instructions. The PI(3,4,5)P3 produced was determined by comparison with a standard curve consisting of PI(3,4,5)P3 standards bound to the ELISA plate. Different amounts of proteins of cell lysates, from which p110δ was immunoprecipitated, were tested in pilot experiments to ensure that the appropriate amount of enzyme were used so that the produced PI(3,4,5)P3 was in the range of the respective standard curve.

### Statistical analysis

Error bars displayed in the figure section represent s.e.m. and were calculated from technical or biological replicates as described in the figure legends and in the description of the respective methods. Data shown are representative of at least three independent experiments, including animal studies, histological images, blots and gels. Data were analyzed using the STATISTICA 7 statistical software package. Statistical significance was determined using the non-parametric Mann–Whitney test; *P < 0.05; **P < 0.01; ***P < 0.001.

## Electronic supplementary material

Supp Figure 1

Supp Figure 2

Supplemental figure legends
