# Supplementary figures and images for: Pharmacological inactivation of the PI3K p110δ prevents breast tumour progression by targeting cancer cells and macrophages
Source: Cell Death Dis. 2018 Jun 7;9(6):678. doi: 10.1038/s41419-018-0717-4 (PMC5992183; doi:10.1038/s41419-018-0717-4)

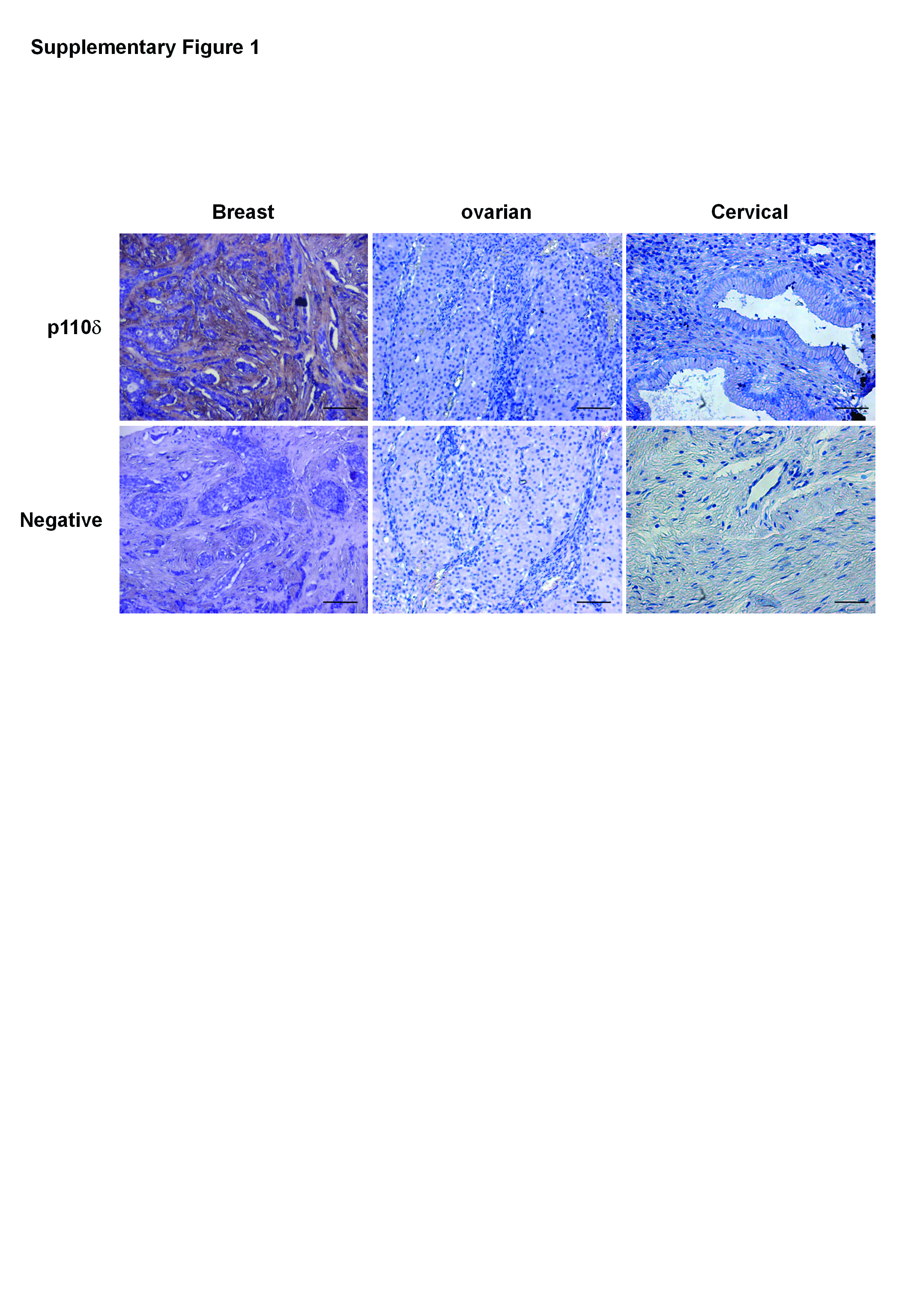

Supplement: Supplementary file 1 — Supp Figure 1 [file 41419_2018_717_MOESM1_ESM.tif]

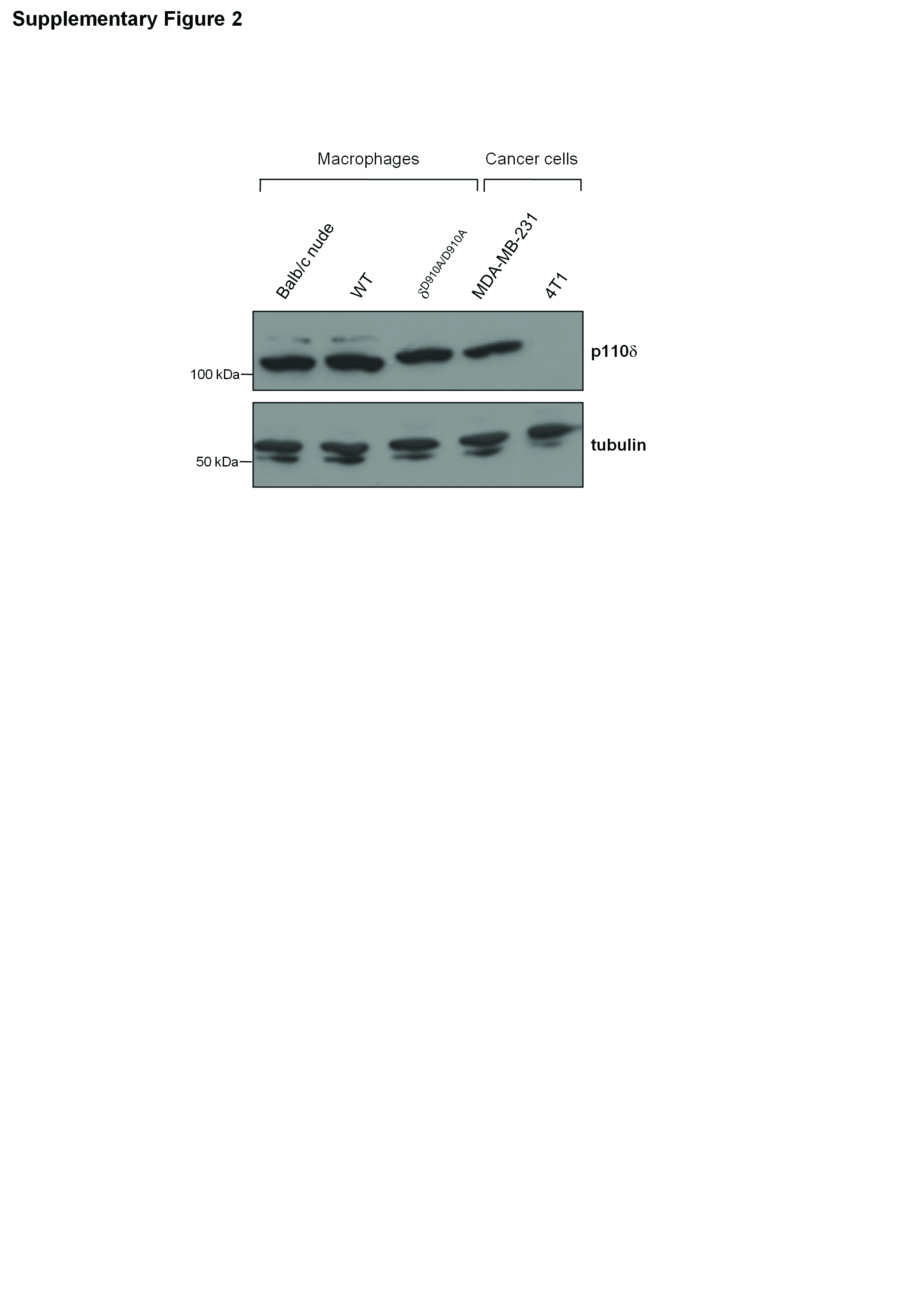

Supplement: Supplementary file 2 — Supp Figure 2 [file 41419_2018_717_MOESM2_ESM.tif]
