# Supplementary material for: Pharmacological inactivation of the PI3K p110δ prevents breast tumour progression by targeting cancer cells and macrophages
Source: Cell Death Dis. 2018 Jun 7;9(6):678. doi: 10.1038/s41419-018-0717-4 (PMC5992183; doi:10.1038/s41419-018-0717-4)
Supplement: Supplementary file 3 — Supplemental figure legends [file 41419_2018_717_MOESM3_ESM.doc]

**[SUPPORTING](http://www.pnas.org/site/authors/procedures.xhtml#si) FIGURE LEGENDS**

**Figure S1 Immunohistochemical expression of the PI3K p110δ in human breast, ovarian and cervical carcinoma tissue.**

Breast, ovarian, and cervical cancer tissue sections were stained for PI3K p110δ. In a control section (negative) for each tissue the primary antibody was substituted by a rabbit IgG. Scale bar= 100 μm.

**Figure S2 Expression of the PI3K** **p110δ in macrophages isolated from mice and in breast cancer cell lines.**

The expression of p110δ was assessed by immunoblotting of total cell lysates (100 μg per lane). The same blot was also immunoblotted using antibody to tubulin as loading control.
